# A Pre-Set Calcium Sulfate/Hydroxyapatite Biomaterial as an Antibiotic-Eluting Bone Extender and a Carrier for BMP-2: A Pilot Study in a Rabbit Posterolateral Spinal Fusion Model

**DOI:** 10.3390/jfb17030118

**Published:** 2026-03-01

**Authors:** Jintian Huang, Gintarė Lukoševičiūtė, Filip Mrkonjic, Hadis Alidadi, Domantas Jakstas, Sujeesh Sebastian, Lars Lidgren, Magnus Tägil, Deepak Bushan Raina

**Affiliations:** 1 Department of Clinical Sciences Lund, Orthopedics, The Faculty of Medicine, Lund University, 22184 Lund, Sweden; jintian.huang@med.lu.se (J.H.); gintare.lukoseviciutee@gmail.com (G.L.); fmrkonjic@gmail.com (F.M.); hadis.alidadi@med.lu.se (H.A.); lars.lidgren@med.lu.se (L.L.); magnus.tagil@med.lu.se (M.T.); 2 Department of Orthopedics and Traumatology, Lithuanian University of Health Sciences, LT-44307 Kaunas, Lithuania; 3 Spinal Neurosurgery Unit, Department of Neurosurgery, Lithuanian University of Health Sciences, LT-44307 Kaunas, Lithuania; jakstas.domantas@gmail.com; 4 Michael Ogon Laboratory for Orthopedic Research, Orthopedic Hospital Vienna-Speising, 1130 Vienna, Austria; sujeesh.sebastian@oss.at

**Keywords:** spinal fusion, bone graft extender, ceramic material, BMP-2, infection

## Abstract

Synthetic biomaterials used as bone graft extenders (BGE) in spinal fusion surgery can supplement but do not replace autologous bone. This pilot study evaluated a calcium sulfate/hydroxyapatite (CaS/HA) material as an antibiotic-eluting BGE and a carrier for bone morphogenetic protein-2 (BMP-2) in a rabbit posterolateral lumbar (L4–L5) spinal fusion model (PLF). Pre-set CaS/HA beads were loaded with tobramycin (TOB) and tested for in vitro antibiotic release and antibacterial activity against *Staphylococcus aureus*. For the in vivo PLF study, CaS/HA beads were used in two treatment strategies: (1) CaS/HA + TOB + autograft (left side) and (2) CaS/HA + BMP-2 (right side). Serum levels of TOB were quantified and spinal fusion was evaluated after 12 weeks. TOB exhibited a rapid initial release, followed by a decline below detectable levels after 6 h in vitro and 48 h in vivo. TOB-loaded CaS/HA beads demonstrated in vitro antibacterial activity for 19 days. In the PLF study, 5/6 and 6/6 specimens were fused radiologically in the TOB and BMP groups, respectively, and 100% using mechanical testing. Micro-CT analysis showed no significant difference in bone volume between the TOB and BMP-2 groups (364 ± 84 vs. 479 ± 95 mm^3^). Histology verified continuous bone bridging in both groups. Our in vitro findings indicate that locally added TOB could protect the CaS/HA material from bacterial colonization and did not adversely impact the CaS/HA material negatively to act as BGE. The addition of low-dose BMP-2 to the CaS/HA material proved effective in building bone without the need to harvest autologous bone. In summary, this pilot PLF study demonstrates that the tested CaS/HA material combined with BMP-2 could replace autologous bone harvesting in spinal fusion surgery. Addition of TOB could potentially protect the material from bacterial colonization during the early post-operative period but further studies in infection models are warranted.

## 1. Introduction

Spinal fusion is a common surgical procedure alleviating pain in various spinal disorders including degenerative disc disease, spinal stenosis and discal herniation [1,2]. While conservative treatment options including physiotherapy and the use of anti-inflammatory/pain medications provide symptomatic relief, spinal fusion surgery is the ultimate treatment option. Refined surgical techniques and advancements in surgical tools for spinal fusion surgery have significantly improved patient outcomes but the treatment is still limited with two major complications. At least 10% of the fusions fail and up to 5% get infected, and often both coincide [3,4,5].

Infection remains the most significant complication that severely impacts healing and clinical outcomes [6,7]. The societal burden of spinal infection is expected to further rise due to the rapidly increasing number of fusion procedures. A nationwide longitudinal database study from the United States during the years 2014 to 2018 has been used to calculate the incidence and payments associated with postoperative infection following spinal fusion surgery. Incremental payments/costs for a patient with deep infection were $74,875 at 12 months, and $93,741 at 24 months [8]. The authors concluded that the implementation of robust evidence-based surgical-care bundles to mitigate postoperative infection in spinal fusion surgery is warranted. Autologous bone harvested from the iliac crest as well as locally from the spine is often used for bony bridging both in posterolateral as well as cage-based spinal fusion procedures [9]. However, in the elderly, who are already at an increased risk of infection, the amount and quality of harvested autologous bone decreases [10]. To overcome these challenges, biomaterials have recently been introduced in clinical practice as bone extenders supplementing autografts. Synthetic bone graft extenders in the form of calcium phosphate and hydroxyapatite biomaterials have been used in various forms including injectable pastes/putties and granules or even sponges [9,11,12]. Although graft extenders reduce the volume of autologous bone needed to harvest, they do not eliminate the need for bone grafting [13]. Furthermore, due to their synthetic origin, they also act as a substrate for bacterial attachment increasing the risk for post-operative infections [14]. We have previously described a biphasic ceramic composite comprising of calcium sulfate (CaS) and hydroxyapatite (HA) as an effective local carrier system for BMP-2, bisphosphonates, and antibiotics in several different models of experimental orthopedic surgery [15,16].

The concept of local drug delivery in orthopedics began in the 1970s with the use of antibiotic-eluting polymeric bone cements [17]. While methacrylic bone cements with antibiotics have been promising, their antibiotic elution relies on passive surface diffusion after which the remaining antibiotics remain entrapped limiting its dosing capability [18]. This shortcoming of bone cement has been overcome with the use of bioceramics consisting of a CaS/HA material eluting gentamicin as the only FDA-approved ceramic carrier in clinical use today. Compared with systemic administration, recent clinical studies have shown that local antibiotic delivery from CaS/HA-based material results in markedly higher drug concentrations at the surgical site, thereby reducing the risk of systemic adverse effects and developing antibiotic resistance [19,20].

Tobramycin (TOB) is an aminoglycoside antibiotic with low lipid solubility and a short half-life, which gives suboptimal concentrations at the surgical site when administered systemically, necessitating high and frequent dosing [21]. In a previous study, we have in vitro demonstrated that TOB premixed with CaS/HA gave a sustained TOB release for up to 35 days, maintaining concentrations above the minimum inhibitory concentration (MIC) [22]. Addition of TOB to the CaS/HA material has not been evaluated in spinal applications to the best of our knowledge. The U.S. FDA, during the last decade, has approved several synthetic bone substitutes to be used as bone extenders, i.e., combined with 50 vol.% autograft [23]. However, bone graft extenders that could mitigate infection during the early postoperative period would be highly beneficial, improving the outcomes of spinal fusion.

Apart from the risk of infection, the other major challenge threatening the surgical outcomes in spinal surgery is the lack of satisfactory bone bridging. We have earlier reported successful bone healing with a low-dose BMP-2 using the CaS/HA carrier [24,25]. By virtue of the unique physicochemical properties of the CaS/HA material, our research group has systematically reduced the BMP-2 doses per volume scaffold by a factor six in a rat critical femoral defect model and a factor 25 in a rat spinal fusion model when compared with the 1.5 mg/cm^3^ dose recommended for the only FDA-approved Medtronic^®^ (Dublin, Ireland) Infuse bone graft [24,25].

To our knowledge, nobody has hitherto studied a biodegradable CaS/HA biomaterial, impregnated with antibiotics and/or BMP, as a bone extender. The combination has the potential to be a groundbreaking step toward robust and fully consistent healing in spinal fusion, eliminating the need for autologous bone graft, simultaneously preventing both infection and non-healing. In this study, a combined approach using pre-set CaS/HA beads impregnated with TOB and BMP was therefore evaluated in a rabbit posterolateral lumbar fusion (PLF) model. This model was described by Boden and co-workers as well as in the ASTM standard F3207-17, and has been recognized by the FDA as an experimental model for evaluating different regenerative medicine strategies for spinal fusion [26,27]. The two aims of this pilot, in vivo study, therefore, were to (1) evaluate the use of TOB-impregnated CaS/HA material as a bone graft extender with systemic TOB pharmacokinetics and impact of TOB on the efficacy of CaS/HA as a bone graft extender; and (2) evaluate the potential of CaS/HA biomaterial combined with BMP-2 as a substitute to autologous bone. The overarching goal of this experiment was to evaluate whether the tested materials achieved fusion outcomes comparable to the current gold standard autologous bone.

## 2. Materials and Methods

### 2.1. Study Design

**Figure 1:**
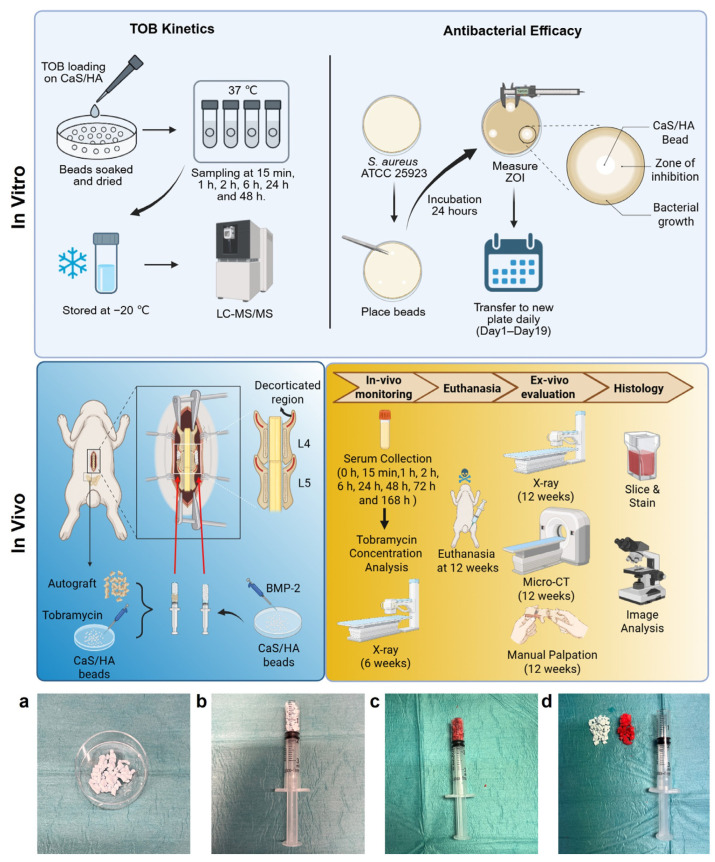
Schematic overview of the in vitro and the in vivo experimental workflow and timeline. (**a**) CaS/HA beads loaded with drugs. (**b**) CaS/HA beads prepared in an open-end syringe. (**c**) Morselized autograft fragments prepared in a syringe. (**d**) Configuration of the material at the time of implantation, with autologous bone placed immediately above the transverse process first, followed by the placement of CaS/HA beads on top. This figure was created using BioRender.com.

The study is divided into in vitro and in vivo parts. The in vitro experiments assessed the TOB release kinetics from CaS/HA beads and their corresponding antibacterial activity. The in vivo experiments employed a rabbit PLF model to evaluate TOB release kinetics in serum and the fusion outcomes of two treatment strategies: CaS/HA + autograft + TOB and CaS/HA + BMP-2. A schematic overview of the study design is presented below (Figure 1).

### 2.2. Materials

Tobramycin (Nebcina, 40 mg/mL; Viatris AB, Stockholm, Sweden) was purchased from the local pharmacy (Apoteket AB, Lund, Sweden). The CaS/HA biomaterial was a gift from Moroxite AB, Lund, Sweden. *E. coli* derived recombinant human bone morphogenic protein-2 (BMP-2) was purchased from GenScript (Biotech Corp., Piscataway, NJ, USA). The standard strain *Staphylococcus aureus* (*S. aureus* ATCC 25923) [28] was purchased from American Type Culture Collection (Manassas, VA, USA). Weigert’s iron hematoxylin was purchased from Sigma-Aldrich (St. Louis, MO, USA). Eosin Y was purchased from Thermo Scientific (Waltham, MA, USA). All other reagents used were of analytical grade unless otherwise specified.

### 2.3. In Vitro Drug Release Profile

To evaluate the release kinetics of TOB from CaS/HA beads in vitro, CaS/HA paste was prepared by mixing 1 g of CaS/HA (60 wt.%/40 wt.%) powder with 0.38 mL of saline. The paste was injected into a hemispherical mold (Ø = 3 mm) to form beads. After setting, beads corresponding to a volume of 1.5 mL (approximately 70 beads) were loaded with 400 µL of TOB solution (16 mg, 40 mg/mL). The solution was applied dropwise onto the beads and allowed to soak and dry at room temperature for 10 min. Six beads were randomly chosen, transferred into individual microcentrifuge tubes containing 1 mL of sterile PBS, and incubated at 37 °C. At predefined time points (15 min, 1 h, 2 h, 6 h, 24 h, and 48 h), PBS supernatants were collected and stored at −20 °C until further analysis. After each collection, the PBS was replaced with fresh pre-warmed PBS. The concentration of TOB released in the PBS solution was subsequently quantified using liquid chromatography–tandem mass spectrometry (LC–MS) by RedGlead Discovery AB, Lund, Sweden. The developed method has a lower limit of detection corresponding to 1 µg/mL.

### 2.4. Antibacterial Efficacy Evaluation

The antibacterial efficacy of CaS/HA composite beads was evaluated against *S. aureus* ATCC 25923 using the Kirby–Bauer disk diffusion assay. Six CaS/HA beads were prepared as described above. Each bead carried approximately 228 µg of TOB and was placed onto Mueller–Hinton agar (MHA) plates. Bacterial suspensions were prepared by resuspending isolated colonies in sterile saline solution (0.9% NaCl, B. Braun, Melsungen, Germany), and the optical density (OD_600_) was adjusted to 0.1 ± 0.005 using a spectrophotometer. The bacterial suspension was evenly spread across MHA plates using sterile cotton buds to establish lawn cultures. CaS/HA beads containing TOB were placed on the surface of the inoculated plates and incubated at 37 °C for 24 h, after which the zone of inhibition (ZOI) around each bead was measured using a standard ruler. After each 24 h incubation, the beads were transferred to freshly inoculated MHA plates containing *S. aureus*, and the procedure was repeated daily until day 19 when no ZOI was observed. Images of ZOI were captured on days 1 to 19 using the ChemiDoc™ MP imaging system (Bio-Rad Laboratories, Hercules, CA, USA).

### 2.5. Animals

Six female New Zealand White rabbits (21–22 weeks old) were obtained from Charles River Laboratories (L’Arbresle, France) and housed at the animal facility of the Lithuanian University of Health Sciences, Kaunas, Lithuania for a period of 7 weeks prior to experimentation. Despite being a pilot study, sample size was calculated empirically based on the recommendations from the ASTM standard and previous studies with similar study aims [27,29]. All procedures involving animals were conducted in accordance with national and institutional guidelines and were approved by the relevant Lithuanian Governmental authority overseeing animal welfare (Permit Number: G2-277). The study followed the Animal Research: Reporting of In Vivo Experiments (ARRIVE) guidelines to ensure transparent and ethical reporting of all in vivo procedures [30]. After 12 weeks of operation, the animals were euthanized, and the spine (from T12 and downward, without cleaning the overlying muscles) specimens were harvested. The collected spine samples were immediately frozen using dry ice and shipped to the Biomedical Center, Lund University (Lund, Sweden), for further analysis. The study was conducted based on the experimental methods and analysis techniques indicated by Boden [26] and co-workers and ASTM standard F3207-17 [27].

### 2.6. Surgical Procedure

All animals were first weighed and sedated via intramuscular injection of Midazolam (5 mg/mL), using a volume of 0.3 mL. General anesthesia was then induced and maintained using a combination of inhalation anesthesia (isoflurane mixed with oxygen) and ketamine (subcutaneous injection, 0.3 mL, 100 mg/mL). Rabbits were positioned in sternal recumbency, and the lumbar vertebrae were localized using a posterior–anterior radiograph acquired with a portable C-arm fluoroscope in the surgical suite. A midline skin incision was made starting at the L3 level and extended 6 cm caudally. To access the transverse processes, a secondary incision was made approximately 2 cm lateral to the midline, followed by blunt dissection of the iliocostalis and longissimus muscles. The transverse processes were exposed and decorticated using a 1.5 mm round burr attached to a motorized handpiece. Decortication was considered complete when punctate bleeding from the bone was observed. Two types of biomaterial grafts were prepared: (1) 1.5 cc of CaS/HA beads loaded with 16 mg of TOB in 400 µL (40 mg/mL) for the left side of the spine; and (2) 2 cc of CaS/HA beads soaked in 100 µg BMP-2 dissolved in 400 µL of saline for the right side. Additionally, 1.5 cc of autologous bone graft was harvested from the iliac crest, morselized into fragments <5 mm using rongeurs, and mixed with 1.5 cc TOB beads on the left (Figure 1a–d). CaS/HA beads were allowed to interact with the TOB and BMP-2 solution for 10 min prior to implantation to enable complete liquid soaking without extending the surgery time significantly. The beads comprised 2–4 mm long cylinders with an approximate diameter of 2 mm and were sterilized using gamma irradiation at 25 kGy. The prepared graft materials were placed to bridge the decorticated transverse processes at the L4–L5 level. In the case of the CaS/HA + TOB group, autologous bone was placed as the first layer above the transverse processes followed by CaS/HA + TOB as the second layer. Muscle and the overlying fascia were closed using resorbable 3–0 vicryl sutures and the skin tissue was closed using staples which were removed within two weeks of surgery. Following surgery, all animals were housed individually, provided with standard rabbit feed, and had free access to water. Postoperative care was conducted according to institutional animal welfare protocols. Animals were culled at the 12-week time point to determine spinal fusion using radiography, micro-computed tomography (micro-CT), mechanical palpation, and histology. The end point of 12 weeks was chosen based on previous studies as well as the ASTM standard [27,29].

### 2.7. Blood Collection and Serum Antibiotic Analysis

Following implantation of the material at the surgical site, blood samples were collected from the ear vein at predetermined time points of 0 h, 15 min, 1 h, 2 h, 6 h, 24 h, 48 h, 72 h, and 168 h. The time was started when the muscle tissue was closed, which was approximately 3–5 min after the material was placed between the transverse processes. The collected blood was immediately centrifuged to isolate serum, which was then stored at −80 °C until analysis. Serum concentrations of TOB were determined using the same method. Prior to analysis, serum samples were processed via protein precipitation using acetonitrile containing cetirizine hydrochloride as an internal standard. TOB levels were then quantified based on calibration curves generated from known standards under identical conditions.

### 2.8. Radiographic Assessment of Fusion

To evaluate spinal fusion, planar radiographic images in the posteroanterior (dorsoventral) projection were acquired using a fluoroscope (Siemens Siremobil Compact L eco, Siemens Healthcare GmbH, Erlangen, Germany) at two different time points (6 weeks and 12 weeks). Further, at the 12-week time point, a high-resolution X-ray image was taken on clinical grade X-ray equipment (DRGEM GXR System) to enable better visualization of the fusion mass. All radiographs were anonymized and independently assessed by three trained observers. Fusion mass was assessed for radiographic fusion on both sides independently. Fusion status was classified as follows: F/F for bilateral fusion, F/NF or NF/F for unilateral fusion, and NF/NF for no fusion on either side.

### 2.9. Micro-CT Analysis

The spine samples harvested at the terminal 12-week time point were imaged using a micro-CT instrument ex vivo. To accommodate the specimen within the sample holder of the micro-CT scanner (MI Labs, Houten, The Netherlands), the spines were trimmed by removing additional vertebrae above and below the L4–L5 fusion segment, such that the scanned region included vertebral levels L3 through L6. Micro-CT scans were performed with the following scan settings: voltage = 65 kV, current = 0.13 mA, total projections = 360 and exposure time of 75 ms. The images were reconstructed to an isotropic voxel size of 30 µm. Reconstructed two-dimensional (2D) slices in sagittal, coronal, and axial planes, along with three-dimensional (3D) renderings of the spine, were independently evaluated by observers to assess fusion status. The same fusion grading criteria used for radiographic evaluation were applied to micro-CT images. For quantitative evaluation, 3D histomorphometric analyses were conducted using Dragonfly software (version 2025.1, Comet Technologies Inc., Montreal, QC, Canada). Parameters including bone volume (BV) and remaining material volume were quantified using Dragonfly. Bone volume and material volume between the right and the left side were compared statistically. Segmentation of bone and high density CaS/HA material in the entire image stack was performed manually by two independent observers. Both grayscale thresholding and even trabecular bone structure were used to separate the bone from the CaS/HA material. A cumulative variance of <15% between the two observers was set as the acceptance criterion. The quantitative measurements from both observers were averaged to present the data on bone volume and material volume.

### 2.10. Manual Mechanical Palpation

Fusion assessment was performed by mechanical palpation by three independent observers on ex vivo spine specimens. All samples were stored frozen immediately after harvest and stored in this state until testing, with no freeze–thaw cycles in between. Prior to palpation, specimens were thawed in a refrigerator at 4 °C for 24 h, followed by an additional 4 h at room temperature to ensure complete thawing. To assess motion at the fusion level (L4–L5), the spine was grasped using the thumb and index finger of both hands—one hand securing the L4 vertebra and the other the L5 vertebra. Gentle force was then applied to induce both lateral bending and flexion–extension movements. A specimen was graded as F (fused) if no motion was detected, and as M (mobile) if any motion was observed during testing. Each specimen was evaluated independently by all observers under blind conditions.

### 2.11. Histological Analysis

Histological evaluation was performed on tissue samples harvested from the L4–L5 fusion segment of all sacrificed animals. Specimens were fixed in 4 wt.% neutral-buffered formalin for 72 h. Prior to fixation, all soft tissues were carefully removed using rongeurs, taking care not to disrupt the fusion mass. The spine was then bisected along the sagittal plane using a 0.4 mm Gigli wire to obtain left and right hemisections. One half of each bisected specimen was subjected to decalcification using formic acid until complete decalcification was achieved. Subsequently, the samples were dehydrated and embedded in paraffin wax following standard histological procedures. Serial sagittal sections of 5 µm thickness were cut using a semiautomated microtome (Thermo Scientific, Waltham, MA, USA) and stained with hematoxylin and eosin (H&E). To ensure comprehensive representation of the fusion zone, sections were collected at three different depths from each sample. All stained slides were digitized using a bright-field slide scanner (Olympus, Tokyo, Japan) at 20× magnification. Histological images were used for qualitative analysis of the contents of the fusion mass as well as for determining histological fusion. Histological fusion was achieved if a bony bridge could be seen covering the space between the L4 and L5 transverse processes. The bony bridge did not require being one continuous structure if the gap between two bone pieces was either filled with bone marrow or the CaS/HA material.

### 2.12. Statistical Analysis

All quantitative data were expressed as mean ± standard deviation (SD) unless otherwise stated explicitly. Statistical comparisons between groups were performed using paired Student’s *t*-test. A *p*-value < 0.05 was considered statistically significant. In addition, 95% confidence intervals (CI) of the differences in means were calculated and reported alongside *p*-values in the Supplementary Materials. All statistical analyses and data visualizations were performed using GraphPad Prism version 10 (GraphPad Software, San Diego, CA, USA).

## 3. Results

### 3.1. Antibiotic Analysis

**Figure 2:**
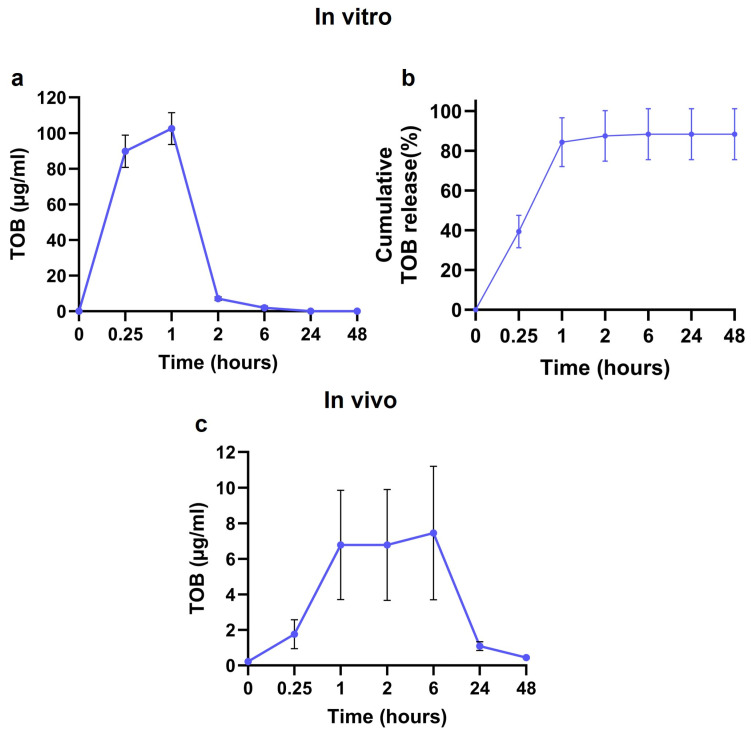
Drug release profile of TOB from CaS/HA beads in vitro and in vivo. (**a**) In vitro release of TOB from CaS/HA beads measured at 15 min, 1 h, 2 h, 6 h, 24 h, and 48 h. (**b**) Cumulative release profile of TOB from CaS/HA beads at 15 min, 1 h, 2 h, 6 h, 24 h, and 48 h. (**c**) In vivo release of TOB from CaS/HA beads measured at 0 h, 15 min, 1 h, 2 h, 6 h, 12 h, 24 h, and 48 h. Data are presented as mean ± SEM. Detailed numerical data are provided in Supplementary Table S1.

In the in vitro release study conducted in PBS, TOB exhibited a rapid initial release during the first 1 h, reaching a concentration of 90 ± 9 µg/mL at 15 min and 102 ± 9 µg/mL at 1 h. This was followed by a decline in release rate over the subsequent time points, with the concentration decreasing to 7 ± 1 µg/mL at 2 h and 2 ± 1 µg/mL at 6 h. The concentration of TOB became undetectable (<1 µg/mL) after 24 h (Figure 2a). More than 80% of the loaded TOB was released from the CaS/HA beads within 48 h (Figure 2b). The in vivo release behavior demonstrated a similar release profile when compared with the in vitro assay. Following implantation, TOB release remained relatively stable during the first 6 h, and a noticeable peak in concentration of approximately 7 ± 3 µg/mL was observed between 1 and 6 h post-administration. After reaching this peak, the amount of TOB detected began to decline progressively from 6 to 48 h (Figure 2c). The concentration of TOB was undetectable (<1 µg/mL) after 72 h.

### 3.2. Antibacterial Efficacy

**Figure 3:**
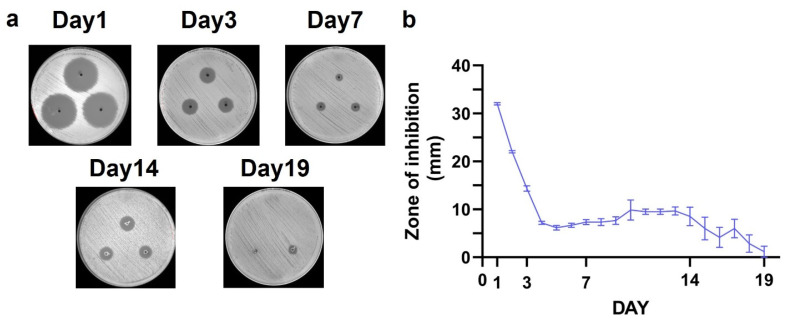
In vitro antimicrobial activity of CaS/HA–TOB beads against *Staphylococcus aureus* ATCC 25923. (**a**) Representative Muller–Hinton agar plates showing zone of inhibition (ZOI) on days 1, 3, 7, 14, and 19. (**b**) Quantitative ZOI measurements over the experimental timeline, illustrating the day-to-day changes in antibacterial activity. Data are presented as mean ± SEM.

CaS/HA beads loaded with TOB demonstrated strong antibacterial activity against *S. aureus* in vitro. On day 1, the ZOI measured over 30 mm, indicating a high initial release of TOB and a potent bactericidal effect. Over the next two days, the ZOI decreased rapidly, reaching approximately 14 mm by day 3. From day 4 onward, the ZOI stabilized at around 6–10 mm, and this residual antibacterial activity was consistently maintained up to day 19 (Figure 3a,b). During this period, the beads were visibly observed to gradually degrade into smaller fragments, which supports the notion that the antibiotic was well incorporated into the material matrix initially, and was subsequently released in a sustained manner as the material underwent resorption.

### 3.3. Rabbit Spinal Fusion Model

#### 3.3.1. Radiographic Fusion Outcome

**Figure 4:**
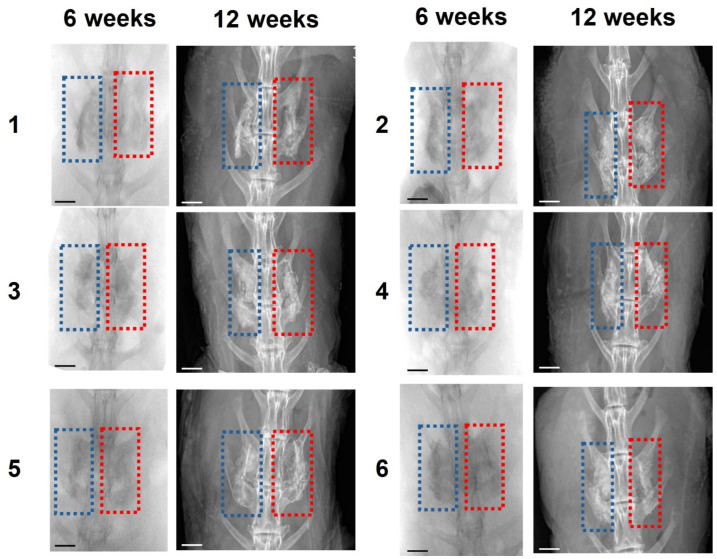
X-ray images of all six rabbits at 6 weeks and 12 weeks. The blue rectangle area within the images indicates the side with CaS/HA + autograft + TOB. The red rectangle area indicates the side with CaS/HA + BMP-2. The scale bar indicates an approximate length of 10 mm. Numbers 1–6 indicate individual specimens analyzed.

At the 6-week mark, five out of six specimens in the TOB group and six out of six animals in the BMP-2 group demonstrated radiographic fusion. Similar results were obtained at the 12-week time point with the BMP-2 group demonstrating a 100% fusion rate at the L4–L5 spinal segment (Figure 4). This outcome was consistently confirmed by three independent observers. Although the BMP-2 group displayed a numerically higher fusion success rate, statistical analysis revealed no significant difference between the two groups.

#### 3.3.2. Micro-CT Findings

**Figure 5:**
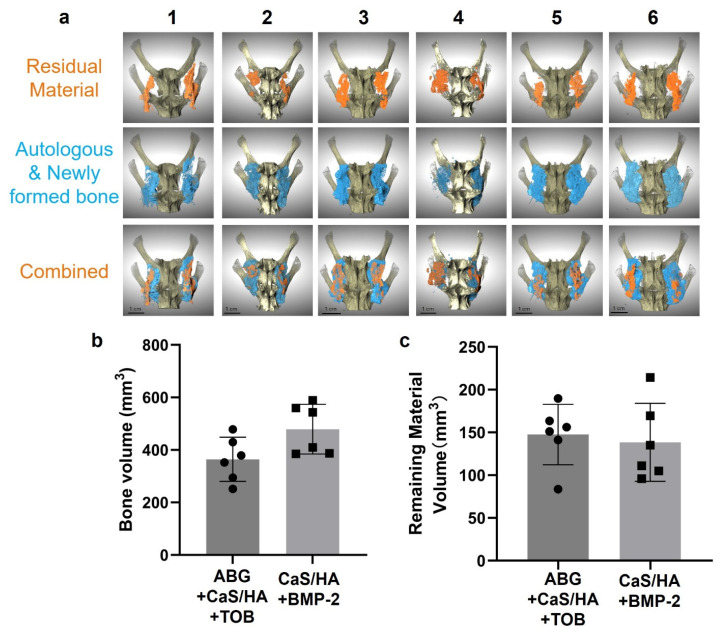
Micro-CT-based evaluation of spinal fusion at 12 weeks. (**a**) Representative 3D reconstructions with orange indicating residual material, while blue represents autologous bone and newly formed bone. (**b**) Quantification of obtained bone volume using Dragonfly. (**c**) Remaining material volume quantified using Dragonfly. Numbers 1–6 indicate individual specimens analyzed.

**Figure 6:**
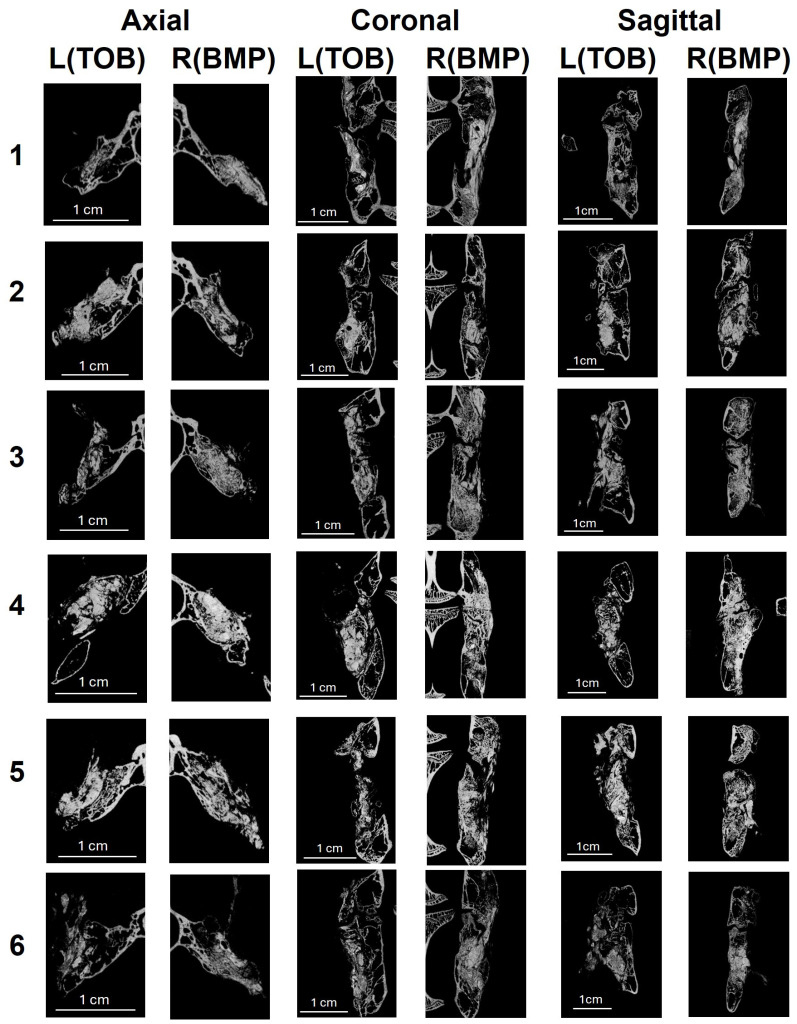
Micro-CT-based evaluation of spinal fusion at 12 weeks. Representative 2D coronal, axial, and sagittal micro-CT slices of the L4–L5 region at 12 weeks indicating the tissue characteristics of the fusion mass. Numbers 1–6 indicate individual specimens analyzed.

Three-dimensional reconstructions were generated and visualized with a full superior view. To complement the 3D visualization, 2D reconstructed slices were also analyzed at four representative levels across the sagittal, coronal, and axial planes for all samples. Based on a comprehensive assessment of both 3D and 2D images, continuous bone bridging across the L4–L5 intervertebral space was observed in all animals from both the TOB and BMP-2 groups, indicating radiographic evidence of successful spinal fusion (Figure 5a and Figure 6). To quantify fusion outcomes, bone volume and remaining material volume were measured (Figure 5b,c). Although the BMP-2 group exhibited a trend toward higher values, no statistically significant differences were found between the two groups for bone volume (*p* = 0.078) or the material volume (*p* = 0.641).

#### 3.3.3. Manual Palpation

The mechanical stability of spinal fusion was evaluated through manual testing by three independent and experienced observers. Each specimen was assessed individually for intersegmental motion, and the final determination was based on the consensus among the three evaluators. All six spinal specimens were classified as mechanically stable, with no detectable motion observed (in flexion or lateral bending) between the L4 and L5 vertebrae (Table 1).

#### 3.3.4. Histological Findings

**Figure 7:**
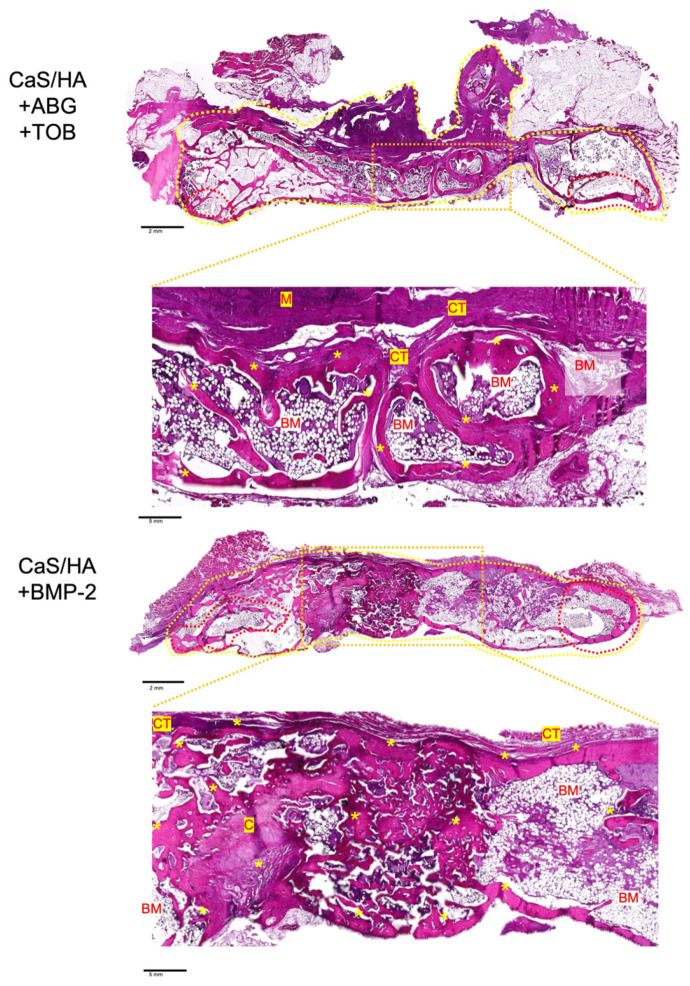
Representative histological images of samples at 12 weeks postoperatively stained with H&E. Bridging cortical bone formation between the L4 and L5 transverse processes is observed in both groups. The dashed red circle indicates the original transverse process, while the dashed yellow circle shows the entire fusion mass including the original transverse processes. The image below the overview images highlights the area covered by the orange dashed box. * Indicates bone, BM = bone marrow; C = cartilage; CT = connective tissue; and M = material.

Histological analysis revealed pronounced new bone formation and the presence of residual CaS/HA material on both the left and right sides of the fusion site. The newly formed bone was observed to bridge the upper and lower transverse processes, forming a continuous bony connection, indicative of successful structural integration. Within the remaining material, cavities and infiltration of granular cells were evident, suggesting active material resorption and ongoing cellular response. When comparing the two treatment groups, a larger area of bone formation was observed on the BMP-2 side relative to the TOB side (Figure 7).

## 4. Discussion

In this study, we used a rabbit posterolateral spinal fusion model described by Boden and co-workers [26] to determine (a) the effect of addition of TOB to a CaS/HA biomaterial’s ability to act as a bone graft extender; and (b) the material’s ability to act as a carrier for BMP-2 and thereby act as an off-the-shelf replacement to autologous bone in spinal fusion. This FDA-recognized animal model is used to evaluate the performance of orthobiologics in posterolateral spinal fusion and provides a fair estimation of device performance in humans. Our study showed that adding a high dose of TOB to a CaS/HA biomaterial did not impact the material’s ability to act as a bone graft extender. The bone-forming potential of this antibiotic-eluting bone extender was in fact at par with a BMP-2-eluting CaS/HA material developed to be an off-the-shelf replacement to autologous bone. A reasonable concordance between in vitro and in vivo TOB elution from the CaS/HA biomaterial was observed. An early antibiotic burst release within the first 1–6 h was observed both in vitro and in vivo, which continued to be detected up to 6 h in vitro and 48 h in vivo. TOB elution from the CaS/HA material continued at levels under the lower limit of quantification for at least 19 days as shown by the inhibition of *S. aureus* growth in the in vitro experiment, indirectly alluding to concentrations ranging between 0.4 (MIC of TOB against *S. aureus*) and 1 µg/mL (LLOD) for 1–19 days.

The FDA, over the last 5 years, has approved ceramic materials to be used as bone graft extenders combined with autologous bone without any known detrimental effects on the biological properties of autograft or fusion outcome. Being a foreign material, bone extenders can however also act as a substrate for pathogenic bacteria to colonize during surgery leading to an infection, severely impacting spinal fusion outcome with severe patient suffering and high societal cost. To mitigate infection, locally added antibiotics can play a pivotal role. If the extender contains an antibiotic, one could theoretically address both shortage of bone grafts and simultaneously prevent infection.

Polymethyl methacrylate bone cements are extensively used in prosthetic joint surgery for localized antibiotic delivery and in combination with systemic antibiotics demonstrated efficacy in reducing postoperative infections. However, only a small antibiotic fraction is released from the acrylate surface within the first few days, followed by subinhibitory concentrations hypothetically claimed to induce bacterial resistance [31]. In contrast, the CaS/HA + TOB combination provided a burst release followed by a shorter sustained release, which is speculated to be advantageous as a prophylaxis and to mitigate bacterial resistance [32]. In a previous study, we have shown that when TOB was pre-mixed with CaS/HA in the paste phase, the release was sustained for up to 35 days, and with complete release observed in PBS under in vitro conditions [22]. In the current study, we impregnated TOB onto preset CaS/HA beads and verified effective impregnation and release induced by gradual material resorption.

Nonetheless, the in vivo environment is markedly different, particularly due to the presence of serum proteins that may passivate the material and impair antibiotic release. In the present study, TOB was investigated as a single agent, and the TOB-loaded CaS/HA beads exhibited a steady release over the first 48 h, with peak concentrations observed between 2 and 6 h. Notably, TOB levels remained above the MIC against *S. aureus* (MIC > 0.2–0.4 μg/mL) at 48 h [33]. Clinically, a single dose TOB administered systemically prior to surgery is considered effective for approximately 24 h. However, due to a short half-life of around 2 h, timing of the preoperative injection is critical and may result in quicker sub-MIC TOB concentrations thereby reducing efficacy and increasing the risk of infection [21]. The in vivo pharmacokinetic profile observed in this study shows that TOB MIC levels can be maintained for at least 48 h, potentially providing effective early-phase protection against infection. It is important to note that the local concentrations of antibiotics in the surgical site will be several orders of magnitude higher than what is reflected by the antibiotic levels in the systemic circulation. This has been shown earlier in a clinical hip fracture study augmented with a similar biomaterial containing a different aminoglycoside, gentamicin-eluting local concentrations reported to be up to 1000 times higher than the serum values [20]. The dose of TOB used in this study equates to 24 h maximum dose TOB given to a human. Our goal was to evaluate whether the addition of a maximum dose TOB added to the CaS/HA material would cause detrimental effects on the material’s ability to act as a bone graft extender and simultaneously protect the material from bacterial colonization. We are currently evaluating lower doses of TOB added to the material to reduce any systemic toxicity, particularly in elderly high-risk patients with reduced kidney function.

In PLF, a large volume of iliac crest graft may be required in addition to locally harvested graft, an issue that becomes even more challenging in osteoporosis and in multilevel fusions, increasing the failure rate [34]. Ceramic materials have been widely used as autograft extenders due to their biodegradability and osteoconductive properties. A previous study has also demonstrated that ceramic materials, when combined with autograft, achieve high fusion rates [35]. In the present study, X-ray evaluation indicated fusion in nearly all samples. However, since ceramic materials can appear radiodense even after 3 months post implantation, radiographic assessment alone may be misleading and could potentially overestimate the actual extent of bone healing. To address this limitation, mechanical evaluation by manual palpation was employed as a supplementary method to verify whether the fusion was both genuine and stable. All samples were verified to be mechanically stable and fused as also verified by Micro-CT and histology, suggesting that replacing up to 50% of the autograft volume with CaS/HA containing TOB may be a viable and effective strategy.

Although CaS/HA has proven osteoconductive properties, its lack of osteoinductivity remains a limitation in regenerating large volumes of bone in a critical bone defect or in a semiorthotopic environment such as PLF surgery. The CaS/HA is a structural scaffold, providing matrix for bone regeneration, but in the case of posterolateral spine, the material requires being combined with autologous bone or even better with BMP-2, as this would completely eliminate the need for bone harvesting from the iliac crest [36]. BMP-2 is one of the most potent osteoinductive cytokines identified to date and is commercially available as part of the Infuse^®^ Bone Graft kit marketed by Medtronic. This medical device has been investigated in spinal fusion procedures to eliminate autologous bone harvesting and minimize donor-site morbidity [25]. The usage of Infuse in spinal surgery has been criticized due to the sub-optimal clinical outcomes and side effects linked to the supraphysiological doses necessitated by the sub-optimal collagen carrier. BMP-2 doses of up to 12 mg/fusion level are commonly employed with the Infuse^®^ product while doses as high as 48 mg have been reported clinically. In terms of BMP-2 concentration/volume scaffold, which provides a volume normalized approach to correlate the efficacy of the carrier, the approved product Infuse^®^ uses 1.5 mg BMP-2/cm^3^ collagen scaffold. To put these BMP-2 concentrations into perspective, we used 50 µg BMP-2/cm^3^ scaffold in this study resulting in a dose reduction by a factor 30. Although we remain judicious in predicting a similar dose reduction in humans based merely on the results from this study, we believe that a reduced BMP-2 dose will eliminate the side effects associated with high dose BMP-2 delivery and secondary rebound osteoclast effect replacing autologous bone harvesting in spine surgery. The possibility of combining both BMP-2 and an antibiotic such as TOB with the same CaS/HA material is now being evaluated. This approach, if successful, holds the potential to be a game changer in spinal fusion surgery.

There are concerns that TOB may exert inhibitory effects on bone healing due to its cytotoxicity to osteoblasts [37,38]. A study by Rousseau M. showed similar periosteal reactions between groups treated with BMP-2 alone and those treated with BMP-2 in combination with antibiotics (tigecycline and tobramycin) [39]. Moreover, Glatt V. et al. also reported that any inhibitory effects of TOB on mesenchymal stem cells cultured under osteogenic conditions could be reversed when used together with BMP-2 [40]. In this study, we applied BMP-2 combined with CaS/HA on one side, and CaS/HA combined with TOB and autograft on the other side. Compared with the autograft healing rate of approximately 70% reported by Crowley J.D., our fusion rate was superior for both combinations of TOB and BMP [41]. Our findings are also in line with other studies evaluating the use of ceramic materials as BGE or BMP-2 carriers in spinal fusion models wherein fusion rates of 80–100% have been reported at the 12-week time point [29,42,43]. In addition, our group has compared pristine CaS/HA as bone extender in another study (unpublished data) with CaS/HA containing TOB (data from this study). No significant differences in CT-based bone formation were observed during the comparison and these findings strengthen the hypothesis that the addition of TOB does not affect the material’s ability to act as a bone graft extender. Furthermore, based on the available data from some of the other FDA-approved bone graft extenders that have been evaluated in the same rabbit PLF model as described in this study, a majority of calcium-phosphate- and hydroxyapatite-based bone graft extenders have shown a healing rate of 70–100% at 12 weeks proving fusion outcomes comparable to autologous bone [29,41,44]. The results obtained in the current study are therefore in line with the results obtained earlier for other approved FDA materials.

There are several limitations in our study. Firstly, only systemic TOB concentrations were measured in the in vivo study due to the pilot nature of this study. We believe that a more comprehensive understanding of TOB pharmacokinetics could be achieved if local TOB concentrations could be studied using micro-catheters. Secondly, TOB and BMP-2 were not incorporated together into the CaS/HA carrier, leaving uncertainty as to whether the pharmacokinetics or the bone-forming effect would differ under such conditions. Further, due to local ethical restrictions, we did not use an in vivo infection inoculation model to directly verify the protective effect of TOB on the CaS/HA material. Despite strong evidence of antibacterial properties of the TOB-impregnated CaS/HA material in vitro, a direct extrapolation of the in vitro results to the in vivo setup must be carefully interpreted until experimental evidence from an in vivo infection model is provided. Moreover, the mechanical testing was performed on an intact spine and not on the left and right segments separately, which was the case for our radiological and histological evaluations. By using the current study setup, we were able to halve the total number of experimental animals in this pilot study, but due to the setup, it was challenging to separate which treatment group contributed to mechanical fusion. Nevertheless, the evaluation of fusion was performed by three other modalities including radiography, micro-CT and histology, all of which showed a comparable outcome between the two treatment groups. Finally, one could argue that this study did not use the autologous bone group alone, nor did we use the pristine CaS/HA material as controls. Autologous bone has been extensively evaluated in this rabbit PLF model and the fusion outcome has generally ranged between 60% and 80%, indicating that the CaS/HA + TOB bone extender achieved fusion rates comparable to autologous bone based on the historical data [29,41,44]. Despite the osteoconductive nature of the CaS/HA material, we have not been able to demonstrate that the material could regenerate large volumes of bone neither ectopically nor orthotopically [15,25]. Considering the pilot nature of this study, we have refrained from using autograft and pristine CaS/HA groups in this pilot study.

## 5. Conclusions

In this study, pre-set biphasic CaS/HA beads loaded with either TOB or BMP-2 demonstrated effective pharmacokinetics during the early postoperative period and supported bone regeneration in a rabbit posterolateral lumbar fusion model. TOB-loaded CaS/HA beads showed substantial antibiotic release both in vitro and in vivo, with sustained in vitro antibacterial activity against *S. aureus* at concentrations exceeding the MIC level. Radiographic, micro-CT, and manual palpation assessments confirmed that local TOB delivery did not compromise spinal fusion, while CaS/HA combined with BMP-2 achieved complete fusion. Histological analysis further demonstrated continuous bone bridging in both treatment groups. Collectively, these findings support the potential of CaS/HA + BMP-2 as an off-the-shelf replacement to autologous bone grafting capable of promoting spinal fusion. Based on the in vitro data against *S. aureus* as well as the in vivo pharmacokinetics data, the CaS/HA material impregnated with TOB could potentially prevent the material from bacterial colonization during the early post-operative days, a concept that was not directly tested in this study. The addition of BMP-2 could transform the osteoconductive CaS/HA material into an osteoinductive matrix capable of guiding successful spinal fusion thereby providing surgeons with a tool to regenerate bone while preventing potential post-surgical infection. Future studies wherein the combination of BMP-2 and TOB is tested in the same CaS/HA matrix are required to elucidate the validity of the concept as a dual drug delivery modality before clinical studies can be initiated.

## Figures and Tables

**Table 1:** Global assessment of spinal fusion at 12 weeks post-surgery. The number of successfully fused specimens out of the total number of specimens evaluated by each assessment modality is shown. The outcomes were consistent among all three independent observers.

Treatment Group	X-Ray	μCT	Mechanical Testing
ABG +CaS/HA +TOB	5/6	6/6	6/6
CaS/HA +BMP-2	6/6	6/6	6/6

## Data Availability

The original contributions presented in this study are included in the article. Further inquiries can be directed to the corresponding author.

## Supplementary Materials

The following supporting information can be downloaded at: https://www.mdpi.com/article/10.3390/jfb17030118/s1, Table S1: Details of Mean ± standard error of the mean (SEM) concentrations of tobramycin (TOB) in vitro and in vivo; Table S2: P-values and 95% confidence interval (CI) for bone volume and remaining material volume between TOB and bone morphogenic protein-2 (BMP-2) groups.
