# Supplementary material for: A Pre-Set Calcium Sulfate/Hydroxyapatite Biomaterial as an Antibiotic-Eluting Bone Extender and a Carrier for BMP-2: A Pilot Study in a Rabbit Posterolateral Spinal Fusion Model
Source: J Funct Biomater. 2026 Mar 1;17(3):118. doi: 10.3390/jfb17030118 (PMC13028491; doi:10.3390/jfb17030118)
Supplement: Supplementary file 1 [file jfb-17-00118-s001.zip › jfb-4169236-supplementary.pdf]

Supplementary Material

Supplementary Text

Antibiotics concentration analysis

Table S1. Details of Mean ± standard error of the mean (SEM) concentrations of tobramycin (TOB) in vitro and in vivo.

Time(h)		0	0.25h	1h	2h	6h	24h	48h
In -vivo	Mean (μg/mL)	0,217	1,7582	6,7852	6,7840	7,4510	1,0835	0,4455
	SEM (μg/mL)	NA	0,814977	3,068393	3,11149	3,748216	0,254137	0,060325
In -vitro	Mean (μg/mL)	0	89,8	102,5	7,1	2,0	0,0	0,0
	SEM (μg/mL)	0	9	9	1	1	0	0

Statistical comparison between TOB and BMP-2 groups

Table S2. P-values and 95% confidence interval (CI) for bone volume and remaining material volume between TOB and bone morphogenic protein-2 (BMP-2) groups.

	P-value	95% CI
Bone volume	0,0783	-18,83 to 248,4
Remaining material volume	0,6413	-56,48 to 38,23
